# The metamorphosis of analytical chemistry

**DOI:** 10.1007/s00216-019-02313-z

**Published:** 2019-12-17

**Authors:** Freddy Adams, Mieke Adriaens

**Affiliations:** 1 Department of Chemistry, University of Antwerp, Drie Eiken Campus, Universiteitsplein 1, 2610 Antwerp, Belgium; 2 Department of Chemistry, Ghent University, Krijgslaan 281-S12, 9000 Ghent, Belgium

**Keywords:** Analytical chemistry, Chemical analysis, Scope, Evolution, Review, Big Data Era, Measurement science

## Abstract

Defining analytical chemistry as the measurement of isolated compositional features in a selected study object ignores the unique perspective that analytical chemists bring to twenty-first century science and society. In this feature article, we will discuss some of the existing preconceptions and misinterpretations of analytical chemistry that occur at present and will tackle them from the more up-to-date perspective of science in the Big Data Era. This will place their influence in context while simultaneously enlarging the scope of the discipline analytical chemistry to its well-deserved prevalent position in present-day science and technology.

**Figure:**
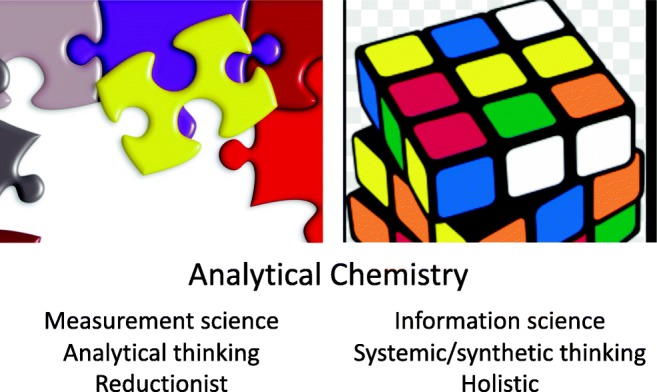
Graphical abstract

Graphical abstract

## Introduction

Over the last 20 years or so, analytical chemistry became increasingly intertwined and embedded as an important component of science in the Big Data Era. Big Data, coupled with new data analytics, challenges well-established concepts in analytical chemistry, engendering paradigm shifts across the discipline. This article examines the consequences of this transformation process in various aspects. The discipline must rethink its fundamentals, its position in science at large, its educational aspects and its practices, rules, and definitions.

Within chemistry, analytical chemistry is the discipline that scrutinises the details of the composition and structure of both natural and man-made objects. It draws upon technological developments and scientific knowledge—both from within chemistry itself and from other scientific disciplines such as physics and biology—to perform its basic tasks, solving particular scientific or technological problems for science and society. As a central scientific discipline, providing many other sciences with information, it delivers spectacularly in the scientific world of today, for example, in the materials sciences and nanotechnology and particularly in the biological and biomedical sciences and biotechnology.

Analytical chemistry has undergone a real “metamorphosis” during the last two decades. In contrast to the word “change”, which implies that some things change but that the essence, the basic principles, remain what they were, “metamorphosis” implies a much more radical transformation, a paradigm shift, in which the old certainties fall away and something quite new emerges. Metamorphosis can be considered as a situation where normal evolution is no longer the case. Under such game-changing conditions, it is necessary to redefine theoretical and conceptual frameworks.

It is, at present, not fully clear how analytical chemistry and chemical analysis should be defined and related to other sciences, particularly to chemistry and metrology. The present situation is compounded by the circulation of distorted, often incomplete and minimalistic, historically influenced views on the essential features of the discipline and on its etymology. Its rules, principles and definitions need to be conceptualised beyond a world of simplified concepts of the past, defining chemical analysis as a single measurement of a given, well-defined chemical substance.

Definitions and rules, methods, procedures and protocols are creations of their time. We need to explore how they evolved from roughly the 1960s to the needs of today. In this paper, we will discuss some of the existing preconceptions and misinterpretations of analytical chemistry that, despite fundamental changes, still apply at present and tackle them from an up-to-date perspective. This will place their influence in context while simultaneously enlarging the scope of the discipline analytical chemistry to its prevalent position in present-day science and technology.

## A brief “analysis” of analytical chemistry

We must revisit the basic concepts before we can move on to a discussion on what analytical chemistry means now and how we should properly interpret its operational characteristics and their meaning in today’s science and technology. This is because analytical chemistry concerns different, quite distinct activities that are not always properly discerned from each other. In order to be able to make reliable conclusions, it is necessary to move back to the basic features of the discipline, but this without commenting or criticising the achievements of the past.

### The anatomy of analytical chemistry

Just like factories producing everything from scratch, from nuts and bolts to complete machineries, including big airplanes (with potentially defective software), analytical chemistry not only encompasses the simple determination of a single constituent. It also encounters situations that require a fully detailed description of the chemical constitution of complex and often highly heterogeneous objects or situations involving multiple related but non-identical objects.

Defining analytical chemistry as the application of compositional chemical knowledge, therefore, ignores the unique perspective that analytical chemists bring to the study of chemistry. The craft of analytical chemistry is not in performing a routine analysis on a routine sample, which more appropriately is called chemical analysis, but in improving established analytical methods, in extending existing analytical phenomena [1].

**Fig. 1:**
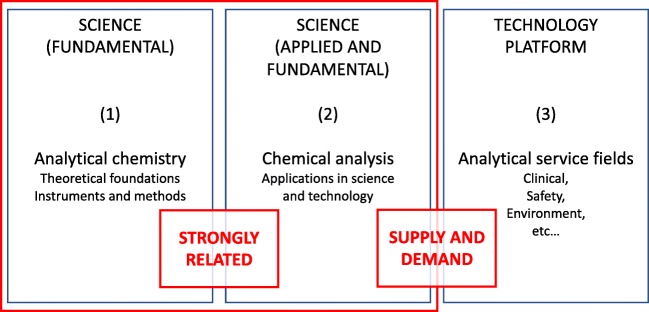
The anatomy of analytical chemistry: (1) analytical chemistry, (2) chemical analysis and (3) analytical services

The realm of analytical chemistry includes producers of science and its users: it has makers and takers. It can be subdivided into three distinct working areas that are summarised in Fig. 1. We discern on the left analytical chemistry as the fundamental discipline (1) within chemistry concerned with the science of measurement of chemical composition. It is the fundamental science of inventing and applying concepts, principles and strategies for measuring the characteristics of chemical systems. It includes the measurement of concentrations but also anything that is important to distinguish an object [2]. On the other hand, there is chemical analysis in the middle of Fig. 1 (2), which involves methods and procedures, ideas, tools and instrumentation developed for application not only in various scientific fields in chemistry but also in other natural sciences and in technological and societal applications. These activities can be fundamental (creating new knowledge) or applied science, helping to understand the complexity of nature or the sophistication of the world of today. Fundamental analytical chemistry and chemical analysis are closely related and sometimes indistinguishable from each other. Furthermore, pure and applied chemical analysis must be clearly distinguished from a third type of chemical analysis-related activity, i.e. analytical services (3): a formalistic, routinely applied technology platform in industry, society and the environment. With analytical services, quality control relies heavily on the use of formal assessment metrics, while the other two items of the triad follow the rules of scientific decision-making processes.

There is a process of supply and demand: activities in chemical analysis and analytical service fields stimulate the development processes activities in (1). Without such input, development of new methodologies might become artificial. Next to that, activities (1) and (2) are discovery driven while activity (3) consists of applications for industry and society.

### Analytical chemistry: how it evolved

Describing the revolution in the life sciences, Sydney Brenner remarked that “students divide history into two epochs: the past 2 years and everything else before that” [3]. In a similar way, seen from a more distant vantage point in time, we can mark analytical chemistry’s rapid development with the changes occurring around the 1960s of the previous century. Before that was the pre-history of the discipline, which was dominated by analytical methods based on the destruction of the sample, chemical separations, equilibrium chemistry and simple instrumental spectrometric tools or electrochemistry. The former dealt mostly in and around the visible part of the electromagnetic spectrum. Nevertheless, many basic concepts developed in that period are still valid today and remain the solid basis of the discipline.

Since the early 1960s, the discipline has been transferred into a new era, often referred to as the Big Science Era, with a large inherent complexity both in the nature and in the number of methodological approaches. The early phase of the explosive expansion of the discipline is, for instance, well illustrated by the monthly editorials of Herbert Laitinen in *Analytical Chemistry*, the journal. The field expanded and increased phenomenally through the introduction of instrumental techniques based on the interaction of radiation and matter, ranging over the electromagnetic spectrum from the terahertz region to hard X-rays and beyond. The use of spectroscopy is ubiquitous as a characterisation tool in many scientific and industrial disciplines. It became possible to relate subtle interactions of radiation with matter to the in situ chemical composition of increasingly smaller objects of study. Various forms of spectroscopy and mass spectrometry (MS) provided major advances in sensitivity, specificity, spatial discrimination (sample size) and speed of analysis with an impressive range of now fully automated analytical instruments.

The resulting full range of analytical methods constitutes a powerhouse of scientific progress. At present, it needs to address problems of growing complexity with methodologies that increasingly need fast repetitive measurements of a widening range of elemental and increasingly complex, often labile, molecular species and structural arrangements and structural defects.

**Fig. 2:**
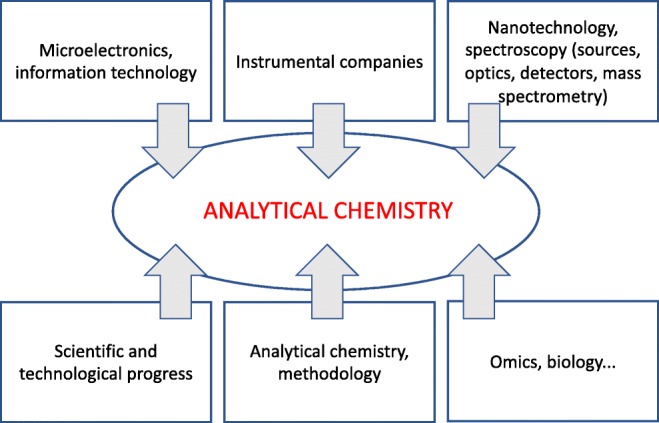
The development of analytical chemistry, tools and ideas, top-down and bottom-up

The phenomenal development of analytical chemistry over the last 50 years was driven both by tools and by ideas [4]. Driving forces came top-down by changes such as microelectronics and information technology, analytical instrumentation companies, micro- and nanotechnology, and bottom-up by general scientific progress in chemistry, biology and physics (Fig. 2).

### The metamorphosis of analytical chemistry

The phenomenal developments seen in analytical chemistry are not uncommon in science. In fact, they are in line with the gradual evolutionary process driven by ideas and tools in many other areas of science [4]. However, what constitutes a real metamorphosis for analytical chemistry is the massive and combined use of analytical instrumentation. It has opened up possibilities to understand complex (natural and technological) heterogeneous materials. It also provides spatial temporal relations between chemical composition, structure and morphology on the one hand and the properties and performance of materials on the other.

**Fig. 3:**
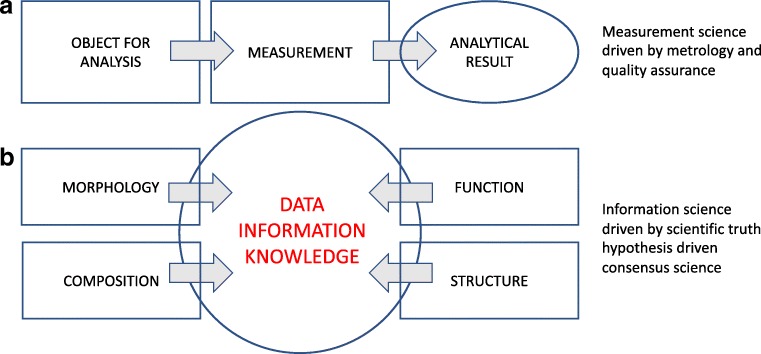
Conventional approach for chemical analysis (a) and approach used in new approaches (b)

This relation is shown in Fig. 3b and is compared with the conventional approach of analytical chemistry in Fig. 3a. It is clear that, in Fig. 3a, the quality of the measurement (metrology, quality assurance) is the most important item of concern, while in Fig. 3b the collected data need to be converted into information to ultimately provide knowledge, and this is driven by information science.

Why this change? In order to understand the link between composition and structure at the nano/microscopic spatial level and the functional behaviour at the macroscopic scale, it became necessary to vastly increase the information acquired from the analytical process. Nanotechnology brought new imaging tools such as scanning tunnelling microscopy, atomic force microscopy and various derived techniques. These are increasingly used as observational tools in combination with spectroscopic chemical imaging analysis and reveal details down to the sub-microscopic level and even to that of individual atoms and molecules.

The focus of many applications in analytical chemistry therefore changed from the singular level in which each analysis concerns a unique compositional entity (Fig. 3a) to the plural, comprehensive level in which analysis concerns the entire detailed composition including structural detail and the interrelation of different components (Fig. 3b).

Overall, the metamorphosis involved the following changes:

- From simple measurements to combinations of tools and techniques (multispectral, hyperspectral, multiplexing of instrumental approaches, compositional relations between many samples, etc.)
- From problem-driven to discovery-driven applications (hypothesis generating)
- Increasingly complex issues for studying nature and materials
- The use of a systemic (holistic) approach rather than one based on unit operations, based on individual measurements

These developments have moved analytical chemistry out of the box, outside of or beyond what is considered usual, traditional and conventional for the evaluation of analytical results. Hence, they have introduced analytical chemistry to information science and into the Big Data Era.

Of particular significance here is the use of the discipline in the characterisation and analysis of micro- and nanostructured heterogeneous technological materials and in various domains of biology including the study of natural objects such as those that evolved from evolutionary processes.

The new concepts in analytical chemistry reverberate the concept of “the gale of creative destruction” coined by Joseph Schumpeter, Nobel Prize in economics for 1993, in which new products and process innovation mechanisms replace outdated ones [5]. However, in our particular case, they do not replace any of the well-established approaches developed in the past. Instead, they add a new dimension and considerably enlarge the scope, the footprint of analytical chemistry in science.

### Analytical chemistry and chemical imaging

For many samples, it is desirable to obtain information about the three-dimensional structure and composition of a particular object, including analytical information as a function of depth, beyond measurement of a 2D surface or a plane within the sample. This can be done by imaging analysis, which is based on the systematic extension from single observations (point, 0-D) to a line (1-D), then to 2-D images on a surface, finally to obtain 3-D information [6]. Chemical imaging is, hence, a significant broadening of spectroscopic analysis.

**Fig. 4:**
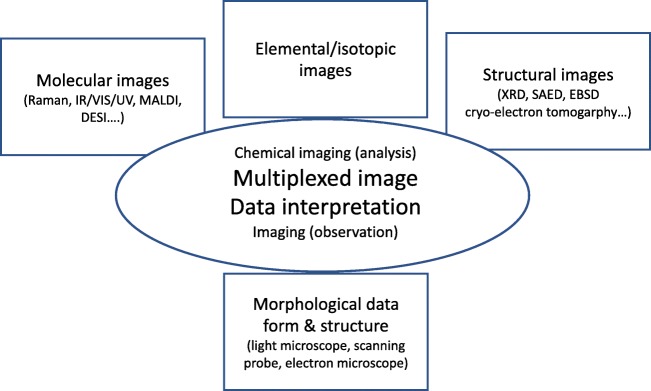
The “chemical analytical microscope”: different characteristics that determine chemical imaging analysis

The overall needs for a full multispectral and multimodal chemical imaging analysis are summarised in Fig. 4. In addition to the chemical imaging methods for elemental information, there are others that provide molecular (the spatial repartition of molecules or functional groups of molecules), structural (the spatial repartition of crystallinity, crystal orientation or phase) or morphological mapping information. Various molecular imaging methods based on IR-VIS-UV observation exist for surface and in-depth molecular characterisation and imaging [7]. Others are based on particle or laser beam interaction with the sample measuring various spectroscopic interaction mechanisms.

**Fig. 5:**
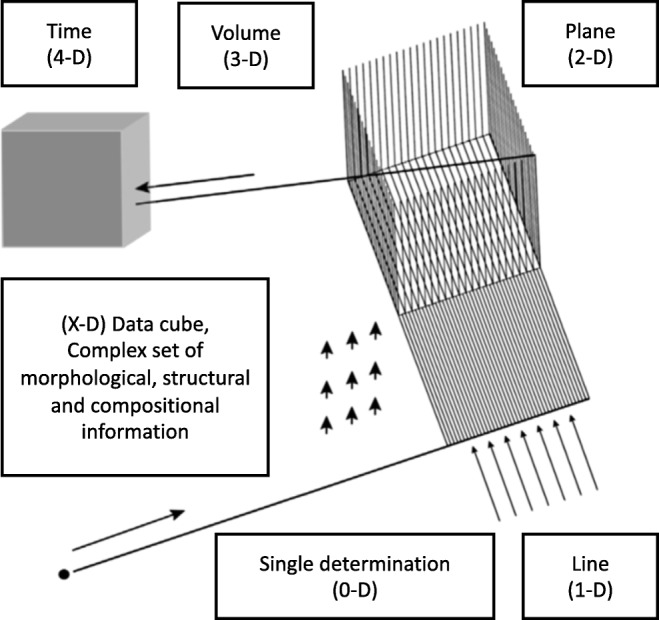
Data structure in multispectral/hyperspectral chemical imaging. From Adams and Barbante [6]; produced with permission

Chemical imaging leads to complex data gathering and interpretation. Figure 5 illustrates schematically the data structure of multispectral/hyperspectral imaging analysis. Every distinct element of a 3D or 4D (for repeated measurements) image can be considered as part of numerical information representative of compositional features in a heterogeneous object. It can also be considerably more complicated when multispectral/hyperspectral data sets result from the combined use of distinct and sometimes orthogonal, observational tools, including density variations (even voids), structural features, changes in morphology or physical properties. In this case, the result is a data cube for every measurement point, with a range of structured information (numbers with their specific significance levels), structured information such as diffraction patterns or high-resolution mass spectra, including sets of collision-induced fragments, morphological information, even textual data, etc. [6].

All this leads to extensive multidimensional data sets, which require deconstruction using various computer manipulation and statistical (chemometrics) tools for obtaining 2D or 3D images for improving the interpretation efficiency. Such chemical data sets are usually quite large. For instance, a state-of-the-art imaging MS data set can easily consist of a pixel set of mass spectra representing the relative abundances of ionised molecules with up to 50,000 *m*/*z* values for medium mass resolution while analysers based on time of flight are able to access *m*/*z* values up to 100,000 and *m*/*z* ratios up to a million or more can be reached with analysers such as Fourier-transform ion cyclotron resonance MS or Orbitrap instruments [8]. The interpretation of the entire set of data and their mutual relation to each other is as important as the metrological quality of the individual measurements.

Many fields of science have undergone a Big Data revolution, including analytical chemistry [9]. The analytical chemistry illustrated in Figs. 3b, 4 and 5 exemplifies its solid incorporation in the Big Data Era and the digital revolution. Large quantities of reliable data originating from natural or man-made objects must be exploited through trustable high-performance computing capabilities and artificial intelligence techniques. The data consist in a cohesive and consistent conglomeration of interrelated and interdependent measurements of individual constituents that are delineated by its spatial and temporal boundaries.

In addition, with the rise in the number of instrumental imaging methods currently available, different analytical methods are increasingly being combined for exploring the same object of analysis. Such a combinational approach is performed either in a single multifunctional instrumental setup or by combining several discrete instruments. Combined imaging methodologies aim at providing the extraction of as much information as possible from a particular sample: elemental, molecular, or other kinds of chemical or physical information, even supramolecular structures with complex architecture. In a targeted approach, one must decide what needs to be measured and the method should be selected and validated before performing the analysis. In a non-targeted approach, the aim is to extract everything feasible from the collected data [6].

Fusion of images consists in merging images together that originate from different modalities in order to create a new hybrid image. Doing this is necessary to keep control of all experimental parameters for reliable and consistent data interpretation. Fusing information across different imaging technologies enables deeper insights and improves interpretation.

### The advent of bioanalytical chemistry

The leading area of development in the “New Age” analytical chemistry is in bioanalysis (molecular biology, biotechnology, pharmacy, medicine). Over the past decade, the advent of microarray technology and robotics has enabled a paradigm shift in molecular biology: a change of emphasis from reductionist approaches and “single-protein” studies for coordinated investigations of increasingly more complex systems of molecules and their interactions and interrelations in space and time. These “systems approaches” (see further) are used to investigate processes as a whole and enable models to be built to predict the behaviour of a system in response to various external cues, disturbances or modifications of its composition [10].

In fact, the importance of biology and biotechnology in analytical chemistry is exemplified by the change of the name of this journal around the turn of the century, from (in German) *Zeitschrift für Analytische Chemie* to *Analytical and Bioanalytical Chemistry*.

Mass spectrometry (MS) is a key contributor in analytical chemistry, particularly for biological applications. An extensive range of MS techniques provides unprecedented capability to identify and specifically determine highly complex compounds with extreme sensitivity at high sample throughput from minute amounts of sample. The development of MS has benefitted from the ability to understand and model ion motion in electric and magnetic fields, and numerous methods for hard and soft ionisation of compounds from complex samples [11]. High-resolution MS also has a unique potential of being able to make use of isotope dilution, a method that is traceable to the fundamental measurement units.

It is essential for many key -omics measurements, such as proteomics, metabolomics, lipidomics and glycomics. Among all these -omic platforms, metabolomics is used to detect the perturbations that disease, drugs or toxins might cause on concentrations and fluxes of metabolites involved in key biochemical pathways. Traditionally, MS-based metabolomics studies can be classified into two primary strategies—a discovery-driven untargeted profiling approach using high-resolution, accurate MS to identify molecular entities, followed by a hypothesis-driven targeted approach to quantify a number of them. The discovery stage detects and identifies potential metabolites that are biologically significant on the level of single cells, while the targeted validation stage can confirm the identity on the basis of fragmentation patterns and quantify these metabolites across large sample populations to enable functional understanding. While the untargeted approach is purely qualitative, targeted analysis can be performed with the highest metrological orthodoxy using isotope dilution MS, a method directly traceable to the SI unit of mass (since the 2019 redefinition of the SI unit of mass with the numerical value of Planck’s constant).

Major obstacles in translating omic data into tangible benefits for practical applications result from the massive high-dimensional nature of the “pan”-omic data. Measuring molecular properties at hundreds of thousands, if not a million or more, features means that associations with outcomes of interest can arise purely out of random chance. In addition, omic data is often plagued by poorly understood sources of data [12]. Advances in computer vision, machine learning and statistical bioinformatics are needed to tackle challenges [13].

**Fig. 6:**
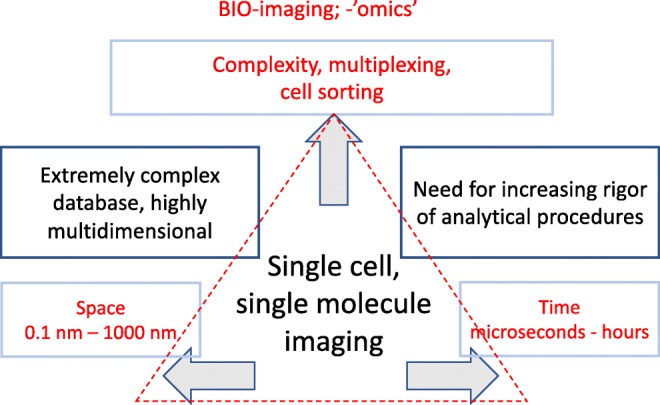
Single-cell pan-omics combining genomics, epigenomics, transcriptomics and proteomics with metabolomics analysis

The emerging field of single-cell characterisation methods addresses fundamental biological questions and allows one to observe metabolic phenomena in heterogeneous populations of single cells. Single-cell metabolomics is an emerging field that addresses fundamental biological questions and allows one to observe metabolic phenomena in heterogeneous populations of single cells. The entire arrangement is schematically represented in Fig. 6. Selected single cells are sorted by cytometric techniques and are subjected to multiplexed and multitechnique analysis. The pan-omics studies incorporate various omics methods with metabolomics analysis. It incorporates genomics (targeting DNA), epigenomics (for DNA methylation and non-coding RNA), transcriptomics (messenger RNA) and proteomics (protein expression) with untargeted metabolomics MS analysis for the detection of small molecules [14]. These studies also tend to incorporate imaging techniques that are capable of tracking the movement of single proteins and molecular complexes in sub-cellular entities visualising the dynamics of a large number of macromolecular assemblies.

The preceding example illustrates the main characteristics of the analytical chemistry of Fig. 3b. First, such experiments produce extremely large multidimensional data collections with many thousands of individual samples each of which giving rise to a highly complex data set. Second, the compositional analytical data are integrated and must be interpreted together with those resulting from the omics platform. Third, the entire study is hypothesis generating rather than hypothesis driven.

Techniques for multidimensional scaling are used to visualise objects as points in low-dimensional metric maps, for increased understanding and visualising similarities in a large number of distinct objects such as cells. These methods are adaptations of principal component analysis, and based on e.g. a quite popular application of stochastic neighbour embedding in a method called t-SNE [15], a methodology particularly well suited for the visualisation of high-dimensional data sets.

Chemical imaging and bioanalysis are, therefore, major areas of development in Big Data science. They produce enormous amounts of data. We should now relate them to the traditional concepts of analytical chemistry, emphasised as they were, and still are, on metrology.

### Analytical chemistry and physics

Many of the new applications of chemical analysis depend on the application of physics. The most spectacular successes of analytical chemistry concern the direct observation of atoms (e.g. with the electron microscope) and molecular arrangements (e.g. with fluorescence mass spectrometry or vibrational microscopies). The highest spatial resolution measurement one can make is at the single atom or molecule level. To determine the concentration of a molecule, the best way is to count the number of molecules in a given volume. As long as the volume contains a statistically large enough number of molecules and is above the Poisson noise limit, counting atoms or molecules is potentially the most accurate (and also the most direct) way to make a measurement [16]. Such analytical chemistry is as good as it can ever get.

One particular example of the influence of physics on analytical chemistry’s development is vibrational spectroscopy. On the macroscopic level, infrared, Raman and fluorescence spectroscopy are quite insensitive methodologies. This is the result of the considerable mismatch between the molecular dimensions and the wavelength of the radiation involved. In 1974, Fleischmann et al. [17] reported strongly enhanced Raman signals from pyridine adsorbed on a silver electrode, an effect called surface-enhanced Raman spectroscopy (SERS). SERS remained analytically non-exploitable for many years because of its erratic nature and this until the detailed mechanisms involved in localised surface plasmon resonance (LSPR) as a function of morphological details were clarified. Currently, a method being used is tip-enhanced Raman spectrometry (TERS), a sensitive molecular detection and imaging tool with a detection limit down to the single-molecule level [18, 19]. Metallic nanoparticles can also be used to increase the sensitivity of fluorescent detection because they generate a phenomenon known as metal-enhanced fluorescence, thus increasing fluorescence lifetime and quantum yields. We identify this revolutionary transformation process, from a scientific curiosity into an ultrasensitive analytical tool, as a metamorphosis.

There are many other such metamorphoses in analytical chemistry. Richard Feynman accurately foresaw many of them in his famous Caltech lecture at that time in 1959: the advent of nanotechnology, the extreme miniaturisation, the direct manipulation of individual atoms and the infinitesimal machinery [20]. On the subject of the electron microscope, he claimed that it would eventually become powerful enough to localise and identify atoms with a precision level of a few picometres (10^−12^ m). He also commented on chemical analysis and its future development in the following prophetic stance: “…physics supplies the foundations of chemistry. But chemistry also has analysis. If you have a strange substance and you want to know what it is, you go through a long and complicated process of chemical analysis. You can analyze almost anything today, so I am a bit late with my idea. But if the physicists wanted to, they could also dig under the chemists in the problem of chemical analysis. It would be very easy to make an analysis of any complicated chemical substance, all one would have to do would be to look at it and see where the atoms are.”

Over recent years, steady advances in transmission and scanning transmission electron microscopy provided the ultra-precise determination of the atomic arrangement of non-periodic structures in materials and the control of nanostructures [21]. This allows for the explanation of various material properties, for example, that strain induced by the lattice mismatch between a substrate and a superconducting layer grown on top can change the interatomic distances by the order of a few picometres and can in this manner turn an insulator into a conductor [22]. Also, as anticipated by Feynman, we may soon have detailed images of life’s complex machineries with atomic resolution. The Nobel Prize in Chemistry for 2017 was awarded for the development of cryo-electron microscopy, which both simplifies and improves direct imaging of the form and detailed structure of individual biomolecules in situ. The detailed structural arrangement of single protein molecules situated at particular locations of a cell membrane can be measured, while even following their structural changes. This new method stands in stark contrast to conventional protein crystallography where it is necessary to crystallise the molecule for the measurement of the collective structure. Would this method eventually be able to replace X-ray diffraction of crystallised material for structural characterisation?

But was Feynman right with his methodologically reductionist and creatively disruptive prediction on analytical chemistry? Of course not, as he explained himself in his reminiscences in his book *Surely you’re joking, Mr Feynman* [23]. The approach based on the direct observation of atomic arrangements with the electron microscope to solve problems in analytical chemistry remains—and will undoubtedly remain in the future—the exception rather than the rule. This is because Feynman reasoned as a physicist while analytical chemistry responds to the fundamental approach of chemistry: analytical chemistry incorporates physics and is not incorporated in physics.

### Analytical chemistry and chemistry

Both physics and chemistry are concerned with matter and its interaction with energy, but the two disciplines differ in approach as they observe the scientific world from different perspectives, from a different viewing angle. In physics, it is typical to abstract from the specific type of matter and to focus on the common properties of many different materials, while chemistry studies the properties of matter with considerably more detail.

Bernadette Bensaude-Vincent, philosopher of chemistry and the author of *Chemistry—the impure science* [24], a book concerned with the philosophical and historic basics of chemistry, expresses this difference as follows [25]: “In optics, for example, materials are characterised by their index of refraction, and materials with the same index of refraction will have identical properties. Chemistry, on the other hand, focuses on what compounds are present in a sample, and explores how changing the structure of molecules will change their reactivity and their physical properties.”

This brings us to the conclusion that analytical chemistry is chemistry, and this not only because chemistry shaped its identity. It is a particular discipline in chemistry; in fact, together with synthesis, it remains one of the basic pillars on which the discipline rests. It scrutinises the details of the composition of natural and man-made objects. It borrows scientific knowledge and technological developments—from within chemistry itself, but also from other sciences such as physics, biology, etc. and from technological developments—to perform its basic task of solving a particular scientific or technical question by using a plethora of different approaches. That is why “analytical chemistry” is now often referred to as “analytical sciences”. As such, it readily incorporates in its realm and embraces, then absorbs, the possibilities offered by nanotechnology and also the electron microscope’s potential of direct observation of atoms rather than being replaced by these developments.

### What is analytical chemistry anno 2020?

Whilst chemistry is the science that looks for the assembly of collections of elementary particles and the plus value it creates with molecular and supramolecular entities, analytical chemistry is the discipline within chemistry that keeps track of these arrangements. This can be done on several levels of complexity, the primary level of compositional details and their quantitative aspects or, in more detail, where are they located, how are they related to each other. This view assumes that analytical chemistry involves scrutiny of everything distinguishing an object, natural or man-made, hard or soft, living or not, on the basis of appearance, structure or properties. In such conditions, the central concept of analytical chemistry and its relation to standard metrological concepts (uncertainty, validation and/or traceability to fundamental standards) seem to lose their central guiding role.

With such an enlarged footprint in science, we can simply summarise analytical chemistry as quoted by Virginia Woolf (of course in an entirely different context):

“I am not one and simple, but complex and many.”

## Discussion

With all this, we can now move to a discussion on how to evaluate analytical chemistry as it has evolved over the last two decades. We will focus on scientific aspects (1) and (2) of Fig. 1 while well realising the importance of the technologic role of the discipline in (3). The new application areas of the discipline discussed in the previous paragraphs belong to systems theory, the interdisciplinary study of systems, to systems thinking instead of analytical thinking. It might be strange to consider analytical chemistry transferring, at least partly, to synthetic thinking modus, but we need to consider its utility in the light of recent developments.

### Analytical chemistry and metrology

When considering Fig. 2a and the prominent place that the measurement is given in the analytical process, it is tempting to consider that (1) analytical chemistry deals with measurements, and (2) since metrology is the science of measurements, (3) analytical chemistry is part of metrology. This (apparently false) application of deductive inference logic leads to propositions to re-baptise analytical chemistry as measurement science, chemical metrology or chemical measurement science [26, 27]. That is also why definitions circulate in many textbooks (and is supported by European learned societies) such as

“Analytical chemistry is a metrological discipline for developing, optimising and applying measurement processes in order to obtain quality (bio)chemical information from natural and artificial systems.”

The situation becomes considerably more complicated for the Big Data situation as in Figs. 2b and 3. The quality of the measurements remains important, but the proper interpretation of the data is compounded by many other sources of error. In a world of increasingly autonomous computer systems, software bugs are a bigger threat than ever before.

While it cannot be denied that metrology formalises the practice of making a reliable measurement, in analytical chemistry and in other scientific fields, such statements constitute—to express it in psychological terms—a cognitive dissonance to us, authors of this review, and probably also to many other analytical chemists.

Just like any other scientific discipline, analytical chemistry must be a faithful user of metrological principles, but not more than that. All this is compounded by the fact that, historically, metrology arose from and is still largely dominated by the definition of measurement units. In practice, requirements for accuracy and precision may vary depending upon the purposes and aims of analysis and the character of the sample [28]. Analytical chemistry and chemical analysis are information related and depend on the rules of scientific research and on the rules and practice of scientific objectivity [29]. This leads to the concept of “consensus values”, with which it is possible to arrive at compliance or agreement between analytical results [30]. This idea, which is also significant beyond the analytical community, points back to the basic philosophical ideas concerning scientific truth such as expressed in science-philosophical treaties by e.g. Karl Popper [31] and science-philosophical concepts such as commensurability introduced by Thomas Kuhn and Paul Feyerabend.

In short, the essentials of science must be brought back in the discipline. In this sense, Jonathan Sweedler advised reproducibility and replicability as basic quality concepts for analytical chemistry, the field, and *Analytical Chemistry*, the journal [32].

A few years ago, the world of psychological research based on functional medical resonance imaging was rocked when an attempt to replicate studies resulted in unsuccessful replications for a significant part of them as a result of the use of defective software for data interpretation [33]. Such a discovery is not unique to psychology. Failure to replicate influential results has also been documented in preclinical cancer research, behavioural social science and experimental economics, among other fields [34]. It is examples like these that lead to ideas that metrology must adapt to the decision processes in the Big Data Era and transform itself into smart metrology, from metrology of instrumentation to the metrology of decision [35].

### Analytical chemistry in the Big Data Era: chemical analysis in the twenty-first century

With the start of the twenty-first century, we have seen the birth of a new industrial revolution, the digital revolution: the ability to store data from various sources (in particular through related items) in unprecedented big quantities and to exploit them through increasingly high computing capabilities using artificial intelligence (AI) techniques and machine learning. In this new scenario called “Big Data”, data reliability becomes an indispensable property: the large amount of data collected and their analysis are completely useless if untrusted information is stored that cannot help to understand a complex reality. In an entirely different context, Rob Kitchin details that Big Data is huge in volume (consisting of terabytes, even petabytes of data), high in velocity (being created in or near real time), diverse in variety (being structured and unstructured in nature) and exhaustive in scope (striving to capture entire populations or systems) [36]. In such “data-driven” research, analytical measurements are performed to generate and confirm new hypotheses rather than to confirm existing ones and use a non-targeted approach. In such conditions, the central concept of conventional analytical chemistry, the analyte and its relation to standard metrological concepts (uncertainty, validation and/or traceability to fundamental constants) seem to lose their central guiding role.

Analytical instrumentation in general and chemical imaging tools in particular follow the concepts of the fourth industrial revolution in integrating different technologies. At its heart, the fourth industrial revolution represents an unprecedented fusion between and across digital, physical and biological technologies, and a resulting anticipated transformation in how products are made and used [37]. As a participating actor, analytical chemistry becomes a partner in a larger scientific and technological aggregate than chemistry.

We need to examine these particular circumstances and see how they affect the fundamentals of analytical chemistry as a scientific discipline.

### Systems thinking versus analytic thinking

In systems thinking, a single measurement becomes part of a conglomerated interrelated entity whose meaning is larger than the sum of its parts. Table 1 summarises some of the most important differences between both thought processes. In brief, analytic thinking is a discriminating process, whereby individual dimensions, concepts and ideas are differentiated from similar ones. In contrast, systemic (or synthetic) thinking is the integrative process of bringing together conceptual dimensions [38].

**Table 1:** Differences between analytical thinking approach and systemic thinking

Analytical approach	Systemic approach
Reductionist approach Emphasis on details Isolates then concentrates on one or more details of the object of interest	Holistic (comprehensive) approach Unifies and concentrates on the interaction between items of study Emphasises global perception
Leads to action programmed in detail	Leads to action through objectives
Possesses knowledge of details, poorly defined goals	Possesses knowledge of goals, fuzzy details

Systems type analytical chemistry acts on the metalevel of the entire experiment instead of concentrating on the details. It is the approach to understanding the larger picture of composition and structure of the object of analysis, rather than the individual measurements, by putting its pieces together. This is in contrast with decades of reductionist views, which involve taking the pieces apart, concentrating on the individual measurements [39]. We summarise the major differences of the two approaches for analytical chemistry in Table 2. Surprisingly, the new analytical chemistry concepts respond to synthetic rather than analytic thinking and for problem-solving concepts.

**Table 2:** Synthetic thinking in “new style” analytical chemistry

Analytic thinking	Synthetic thinking
Measurement science Focuses on measurement uncertainty Metrology is dominant factor	Information science Focuses on scientific consensus building Accepts certain levels of uncertainty Scientific truth, falsifiability
Favours homogeneity Heterogeneity is a source of analytical error	Focuses on heterogeneity It is a study object of natural and technological objects
Leads to discipline-oriented education and method-based organisation	Leads to multidisciplinary education and problem-oriented organisation

### Is present-day analytical chemistry still part of chemistry?

Science is not subdivided in separate disciplines by any natural law, but for practical reasons, and chemistry is one of those disciplines. But is analytical chemistry still chemistry in its present state of development? This question has important repercussions and caveats that will be discussed in the following three sections of this paper.

We need to address a central item of concern: what is the core business of analytical chemistry today? Should analytical chemistry research and analytical chemistry education be organised from within the chemistry departments in the universities or research centres? There is no straightforward answer to all these questions, but they are worth a thorough debate.

### The etymology and semiotics of analytical chemistry

We already discussed that analytical chemistry cannot be identified with metrology or with physics. It is often argued that the name “analytical chemistry” does not cover the subject of the discipline anymore and that it is a name that dates from the period when analytical chemistry was not much more than a collection of wet-chemical methods of analysis. Such a name may be considered as a historic artefact, in the same way as painting schools such as “Flemish Primitives” or “Pre-Raphaelites” are historic names that do not describe their real content as an accurate description of a specific art form.

The Flemish Primitives were not primitive painters in the fifteenth century; on the contrary, they were at the forefront of artistic and technical development at that time. The Pre-Raphaelites, equally, did not originate and work before Raphael; instead, they were a group of British painters that brought an innovative twist to artistic expression in the second half of the nineteenth century.

At present, nobody really minds these apparently completely false denominations. We can argue along similar arguments for analytical chemistry, we can just wait a while and be patient until analytical chemistry as a terminology will get a meaning far beyond its present, literal connotation. In fact, this situation is already the case.

The linguistics behind a name intersects with philosophical considerations. A proper name should refer to a specific object, but specific names may also refer to a more collective whole. This happens with the semantics of analytical chemistry. The discipline is, at present, a broad aggregate of methodologies that derive not only from chemistry but also from physics and biology. Of particular significance is the study of the evolutionary processes that, over the past 2 billion years, produced many potentially important materials. But, increasingly, it has also derived from technological areas and how they will fulfil the needs and expectations of the complex society of tomorrow in a sustainable way [40].

Re-baptising the discipline into “analytical science” or “analytical science and technology” might have some advantage as this terminology encompasses the essential activity—it concerns analysis and the development and application of analytical science—and puts it in a broader scientific context. But what’s in a name? A rose by any other name will smell as sweet…

We keep believing, however, that analytical chemistry remains the right name as it links the discipline where it belongs: chemistry. The highest quoted journal in the discipline is and remains labelled and identified as *Analytical Chemistry*; it covers a lot more than chemical research and nobody seems to mind. The official IUPAC definition of analytical chemistry starts as follows: a scientific discipline that develops and applies methods, instruments and strategies to obtain information on the composition and nature of matter. This definition passed though countless committee meetings, and nobody seems to object to it.

Maybe, all things duly considered, the best is to avoid any definition of analytical chemistry, adhering to the age-old adage that says simply: “Analytical chemistry is what analytical chemists do”.

### Analytical chemistry in education

Two of the core principles of general scientific practice are quantification and systematisation. In that respect, students are taught already early on in their curriculum about the importance of measurement uncertainty and reproducibility of results. It is therefore surprising that, in various disciplines, particularly within the natural sciences, relatively simple or outdated methodologies are being used for quantifying data and for testing physical theories.

The same applies with the chemistry curriculum. Systematics are introduced with the apparent simplicity of the periodic system and then further developed with the intrinsic complexity of organic chemistry and its nomenclature and synthesis. Quantitative aspects of the discipline are introduced with elementary aspects: equilibrium chemistry concepts introduce quantification in the discipline. Important as these deep-rooted principles may be in the education of a chemist, they also provide an outdated view of the discipline. When in most textbooks this is supplemented with instrumental chemical analysis, treatment is fragmental with a deep division between organic and inorganic analysis and a strict separation between the different methodologies. Overall, the measurement and its accuracy dominate the picture. Largely missing in the average textbook is the new holistic synthetic approaches for problem solving described in the previous paragraphs of this paper.

At present, numerous educational initiatives are taken for re-enforcing the analytical chemistry curriculum, and this both on the conventional chemistry and measurement science level of Fig. 3a, as on the multidisciplinary, information science level of Fig. 3b. The Erasmus Mundus Master programme EACH (Excellence in Analytical CHemistry), for instance, remains largely on the monodisciplinary (chemistry) level and on measurement and metrology [41]. The SALSA (the Graduate School of Analytical Sciences Adlershof) Graduate School initiative, on the other hand, offers structured multidisciplinary research combined with an integrated curriculum in analytical sciences [42]. The initiative is carried out at the Humboldt University in Berlin and follows the open-mind integrative concepts of Alexander von Humboldt [43]. Both of these approaches have their pros and cons for educating the young analytical chemists for the complex tasks ahead of them.

In general, we believe that educational efforts in analytical chemistry should focus on the participation, as chemists, in cross-disciplinary and interdisciplinary teams rather than on breaking down the disciplinary border and leaving chemistry.

### The prominent place of analytical chemistry in science

According to Miguel Valcárcel, analytical chemistry is not optimally perceived, and even treated as a second-class discipline. He attributes this to long-lasting prejudices blaming analytical chemists, chemists from other disciplines and even professionals from other fields [44]. We believe that such negative views, if they really exist somewhere, reside within the analytical chemistry community itself and are the result of a lack of self-esteem. The discipline is not a Cinderella suffering from undeserved neglect but a scientific powerhouse, a mighty source of influence and inspiration.

The professional success of analytical chemistry graduates is obvious. It has been demonstrated that there is a shortage of well-educated analytical chemists in Europe [45]. A considerable fraction of the European Research Council grants of the last years highlights innovative research projects linked with chemical analysis or analytical methods development. Table 3 shows a brief selection of recent Nobel Prizes and the place of chemical analysis in them. Striking is the place of bioanalysis and the nano-size level in all this. The particular place of analytical chemistry in the 2018 Physics Prize is documented by Asplund et al., in a recent article in this journal [46]. The importance of lasers and tools for manipulating nano-size samples is obvious. That the other two examples belong to the field of analytical chemistry might be a matter of debate, but we strongly believe they do. The 2014 Chemistry Prize deals with detecting and tracing in space of single biomolecules in order to study their interaction processes, while the 2018 Chemistry Prize involves the measurement of the detailed structural arrangement of single protein molecules in situ in particular cells.

**Table 3:** Recent Nobel Prizes connected with analytical chemistry

Year	Discipline	Awarded to	Motivation
2014	Chemistry	Eric Betzig, Stefan Hell, William Moerner	Super resolved fluorescence microscopy
2017	Chemistry	Jacques Dubochet, Joachim Frank, Richard Henderson	Cryo-electron microscopy for high-resolution structure determination of biomolecules in solution
2018	Physics (joint)	Arthur Ashkin	Optical tweezers and their application to biological systems
	Physics (joint)	Gérard Mourou, Donna Strickland	Method for generating high-intensity ultrashort laser pulses

## Conclusions and outlook

At present, analytical chemistry acts at the interplay between composition, structure and structural defects on one side and properties and functionality of soft and hard material objects on the other. It moved away from a chemistry-oriented field of research to one in a more broadly defined cross-disciplinary denominator, similar to auxiliary fields such as spectrometry, materials science or environmental science. While chemistry remains the basis for its development aspects, its applications in chemical analysis range over the entire scientific realm. Analytical equipment and analytical methodologies are enabling technologies for science and society. In a way, analytical chemistry is like an expat, a migrant (not a fugitive): it has its roots in chemistry, but its ambitions and expectations are situated elsewhere, all over science, in industry and society.

The “new” Big Data Era analytical chemistry is not replacing the traditional concepts of analytical chemistry; it is an epitome, an avatar, a new conceptual form. It still needs to be fully integrated into the conceptual framework of the discipline. In the Big Data Era applications, it cannot rely anymore on the aims and ambitions based on the quality assurance of the individual measurements. Instead, its ambitions must be based on the relation of many analytical results in combination with other relevant information. The combined data are greater than the sum of its parts, because the way they combine adds a different quality. This is an age-old concept supposed to originate with Aristotle.

Central to the full acceptance of the discipline in its new form is how it manifests itself in its practical utility, in both society and science. Big Data analytical chemistry cannot rely on human judgment. Humans are susceptible to cognitive bias; they have a tendency to seek information that confirms their prior beliefs. Also, the application of AI is not without risk [47]. In a world of increasingly autonomous computer systems, software bugs become a bigger threat than ever before. There is a lot of fundamental work to do in ensuring the quality of data collection, data handling and data reduction. The use of chemometrics is necessary to transform data in actionable insight. Eventually, “smart metrology” will play a role in all this.

Only time will tell when it opens its curtains how the discipline will eventually evolve and which new, now totally unexpected metamorphoses it will undergo in the long run.
